# Neurovascular Involvement in Arterial Tortuosity Syndrome Associated with a Homozygous *SLC2A10* p.(Trp162Ter) Variant: Clinical, Molecular, and In Silico Characterization

**DOI:** 10.3390/ijms27156806

**Published:** 2026-07-29

**Authors:** Serdar Bozlak, Cuneyd Yavas, Evrim Yalcin, Yusuf Seflekci, Tunay Dogan, Abdulilah Ece, Nazli Gulsum Akyel, Adnan Yuksel

**Affiliations:** 1 Department of Molecular Biology and Genetics, Faculty of Engineering and Natural Sciences, Biruni University, Istanbul 34015, Turkey; serdar_bozlak@hotmail.com (S.B.); 240407038@st.biruni.edu.tr (E.Y.); yseflekci@biruni.edu.tr (Y.S.); 2 Department of Pediatric Genetics, Basaksehir Cam and Sakura City Hospital, Istanbul 34480, Turkey; 3 Department of Medical Laboratory Techniques, Istinye University Vocational School of Health Care Services, Istanbul 34100, Turkey; tunay.dogan@istinye.edu.tr; 4 Department of Medical Biochemistry, Faculty of Medicine, Biruni University, Istanbul 34460, Turkey; aece@biruni.edu.tr; 5 Department of Pediatric Radiology, Basaksehir Cam and Sakura City Hospital, Istanbul 34480, Turkey; nazligulsumakyel@gmail.com; 6 Department of Pediatrics, Faculty of Medicine, Biruni University, Istanbul 34010, Turkey; ayuksel@biruni.edu.tr

**Keywords:** arterial tortuosity syndrome, *SLC2A10*, GLUT10, molecular docking, molecular dynamics, transmembrane helices, neurovascular involvement

## Abstract

Arterial Tortuosity Syndrome (ATS) is a rare autosomal recessive connective tissue disorder caused by pathogenic variants in *SLC2A10*, which encodes the facilitative glucose transporter GLUT10. Although its vascular features are well recognized, the molecular consequences of many truncating variants remain poorly understood. We report a patient with ATS carrying a homozygous nonsense variant, c.485G > A (p.Trp162Ter), identified by whole-exome sequencing. Quantitative real-time PCR assessed *SLC2A10* expression, and integrated bioinformatic analyses (structural modeling, druggability prediction, transmembrane topology, molecular docking, and molecular dynamics) explored its structural impact. The patient presented with severe systemic arterial tortuosity, congenital cardiovascular anomalies, hernias, connective tissue abnormalities, and neurovascular involvement involving cerebral tortuosity and distal intracranial narrowing. Structural modeling revealed extensive truncation of GLUT10 and loss of multiple α-helical domains, with transmembrane helices reduced from twelve to five. Docking of nine known ligands showed weaker binding to the mutant, and Compound 892 bound most strongly to the wild type (−7.469 kcal/mol). Across 300 ns simulations, the mutant complex proved markedly less stable. qRT-PCR showed no significant transcript differences among patient, carriers, and controls. Our findings broaden the neurovascular spectrum of *SLC2A10*-related ATS and demonstrate that p.(Trp162Ter) severely disrupts GLUT10 architecture, topology, and ligand binding.

## 1. Introduction

Arterial tortuosity syndrome (ATS) is a rare autosomal recessive connective tissue disease characterized by elongation, tortuosity, stenosis, and aneurysm formation affecting large and medium-sized arteries [1]. Patients with arterial tortuosity syndrome (ATS) may present with vascular instability, craniofacial dysmorphism, arachnodactyly, diaphragmatic defects, hyperextensible skin, and connective tissue abnormalities. Severe cerebrovascular and cardiovascular complications remain the major causes of morbidity and mortality in affected individuals. Although ATS is considered extremely rare worldwide, the increasing numbers of molecularly confirmed cases have improved the understanding of disease pathogenesis and genotype–phenotype correlations.

The disease is caused by pathogenic variants in the *SLC2A10* gene located on chromosome 20q13.1. *SLC2A10* encodes GLUT10, a facilitative glucose transporter belonging to the GLUT family of transmembrane proteins. GLUT10 contains 12 transmembrane helices and functions primarily within the endoplasmic reticulum membrane rather than the plasma membrane [2]. This intracellular localization is critical because GLUT10 is responsible for transporting dehydroascorbic acid into the endoplasmic reticulum lumen, thereby contributing to collagen and elastin maturation processes. Loss of GLUT10 activity impairs extracellular matrix organization and weakens vascular connective tissue integrity.

Recent experimental studies additionally demonstrated mitochondrial dysfunction, oxidative stress abnormalities, and reduced oxygen consumption rates in *SLC2A10*-deficient mouse models [3]. These findings suggest that GLUT10 deficiency affects not only extracellular matrix homeostasis but also intracellular metabolic regulation. Therefore, ATS pathogenesis appears to involve both structural connective tissue defects and mitochondrial dysfunction.

Although multiple *SLC2A10* variants have previously been reported, the structural consequences of many truncating variants remain poorly characterized. Furthermore, genotype–phenotype correlations in ATS are still limited due to the rarity of the disease and the small number of structurally investigated variants described in the literature. Herein, we report a novel homozygous *SLC2A10* c.485G > A p.(Trp162Ter) nonsense variant identified in a patient with clinical findings compatible with ATS and investigate its structural and molecular consequences using integrated bioinformatic analyses.

## 2. Results

### 2.1. Literature Search, Case Selection, and Ethics

Cases without reported *SLC2A10* variants, without relevant neurovascular information, or representing duplicate reports were excluded. Extracted variables included demographic data, neurological and vascular manifestations, intracranial/cerebrovascular imaging findings, additional ATS related features, *SLC2A10* variant nomenclature, variant type, zygosity, clinical significance, and reference. Current ClinVar clinical significance was recorded using VarSome searches based on the *SLC2A10* transcript NM_030777.4. The literature derived cases were reviewed together with the present case and summarized in Table 1 to contextualize the neurovascular spectrum associated with *SLC2A10*-related ATS.

### 2.2. Case Presentation and Clinical Findings

**Figure 1:**
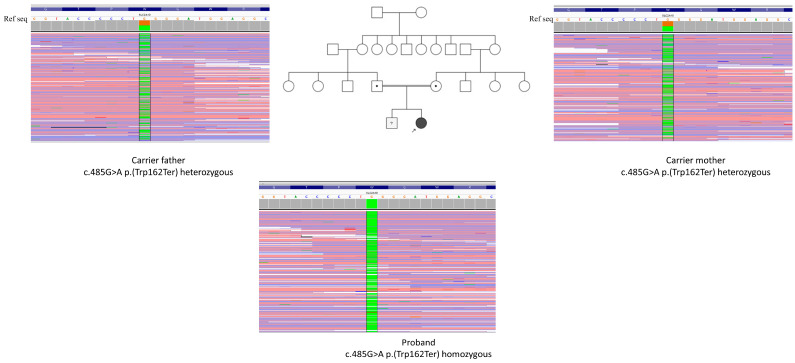
Pedigree and segregation of the next generation sequence images of the family. Squares indicate males and circles indicate females. The filled symbol indicates the affected proband, which is marked by an arrow. Dotted symbols indicate heterozygous carrier parents. The double line denotes parental consanguinity. The symbol with a question mark indicates a sibling with congenital heart disease and unavailable molecular status (Green: Adenine nucleotide; Orange: Guanine nucleotide.

We present a female patient referred for evaluation because of a family history of congenital heart disease and prenatal detection of a ventricular septal defect. The family history was notable for a sibling who died with tetralogy of Fallot. The patient was the first female child of first-degree cousin parents. A three-generation pedigree demonstrated parental consanguinity, with the affected proband indicated by an arrow (Figure 1).

**Figure 2:**
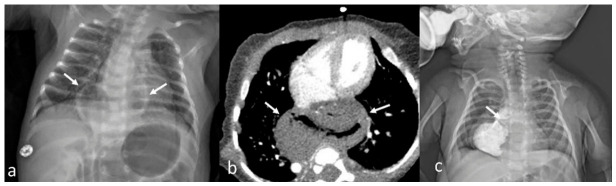
Radiological findings of hiatal hernia at 1 month of age. Posteroanterior chest radiograph demonstrates radiolucent gastric air located superior to the diaphragm and posterior to the heart (**a**). Axial computed tomography image shows findings consistent with hiatal hernia (**b**). Fluoroscopic examination shows the esophagogastric junction at the T7–T8 level, slightly to the right of the midline, with the gastric cardia and fundus positioned above the diaphragm, consistent with a type III hiatal hernia (**c**).

The patient was born at term, at 38 + 3 weeks of gestation, by spontaneous vaginal delivery. Birth weight was 2.9 kg (−0.47 SDS), length was 47 cm (−1.07 SDS), and head circumference was 35 cm (0.65 SDS). There were no postnatal adaptation problems. On neonatal examination, she had dysmorphic facial features, including a long face, long philtrum, micrognathia, and a high-arched palate. Increased skin elasticity and joint laxity were also noted. A posteroanterior chest radiograph obtained at 1 month of age showed radiolucent gastric air located above the diaphragm and posterior to the heart, consistent with a hiatal hernia. Axial CT and fluoroscopic evaluation further supported the diagnosis of a type III hiatal hernia, with the esophagogastric junction located at the T7–T8 level and the gastric cardia and fundus positioned above the diaphragm (Figure 2).

**Figure 3:**
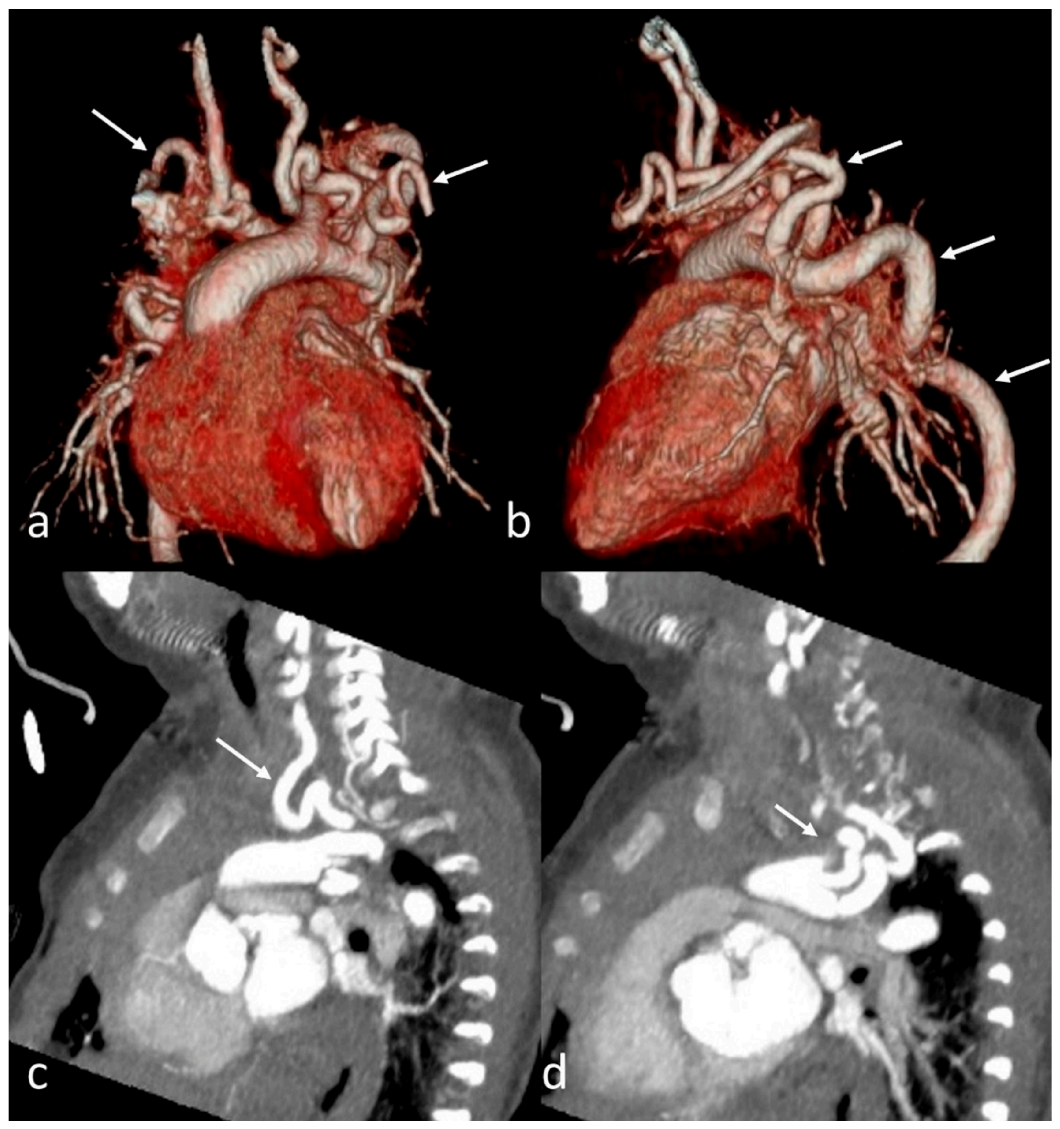
Cardiovascular computed tomography angiography findings at 1 month of age. Cardiac volume-rendered computed tomography angiography images demonstrate severe tortuosity of the aorta (**a**,**b**). Sagittal oblique contrast-enhanced computed tomography images show tortuosity of the right brachiocephalic artery, as well as the right and left common carotid and subclavian arteries (**c**,**d**).

Echocardiography performed at 1 month of age revealed a tortuous aortic arch, ventricular septal defect, peripheral pulmonary stenosis, patent foramen ovale, and persistent left superior vena cava. CT angiography demonstrated severe tortuosity of the aortic arch involving all major branches and the descending aorta (Figure 3).

**Figure 4:**
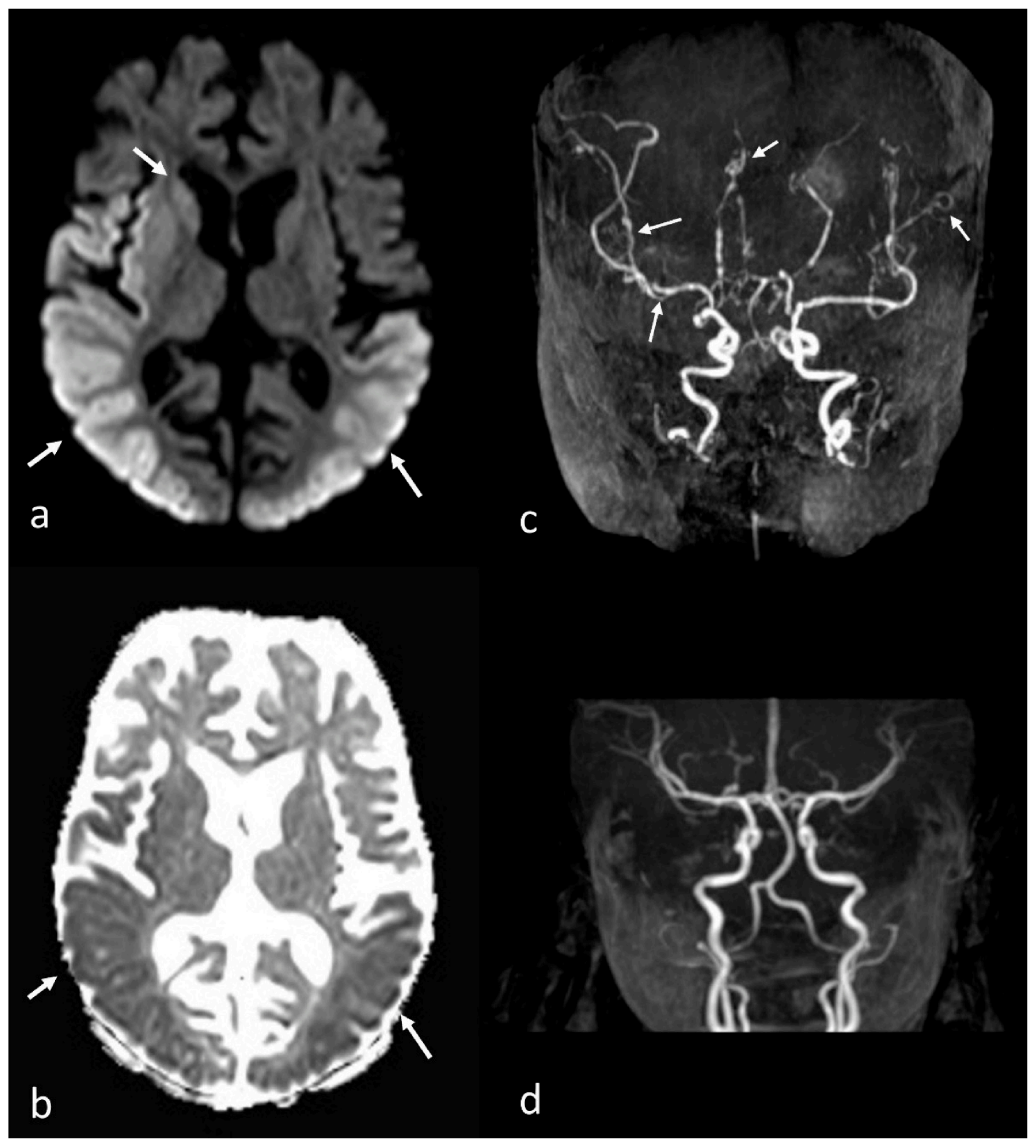
Cranial magnetic resonance imaging and angiography findings at 12 months of age. Following hiatal hernia repair, the patient developed postoperative neurological deterioration. Axial diffusion-weighted imaging (**a**) and apparent diffusion coefficient map (**b**) demonstrate diffusion restriction involving the bilateral cerebral cortex and right striatum, compatible with hypoxic–ischemic encephalopathy. Time-of-flight magnetic resonance angiography with maximum intensity projection reconstruction shows cerebral vessel tortuosity and distal branch narrowing in the middle cerebral artery, anterior cerebral artery, and posterior cerebral artery territories (**c**). Magnetic resonance angiography of an age-matched healthy child is provided for comparison and demonstrates normal vascular morphology (**d**).

Superficial ultrasonography performed for bilateral inguinal swelling showed bilateral inguinal hernias containing ovarian tissue. During early infancy, neurodevelopmental milestones were initially appropriate. The patient achieved full head control, independent sitting, and independent walking by 12 months of age. Social interaction and communication were also considered age appropriate, with sustained eye contact, vocalizations, laughter, and initial simple words. The patient underwent hiatal hernia repair at 12 months of age. Postoperatively, she developed neurological deterioration with loss of previously acquired developmental skills. This was characterized by limited eye tracking, loss of expressive smile, and the inability to sit independently. Subsequent cranial imaging showed diffusion restriction involving nearly all the cortical structures on diffusion-weighted MRI, compatible with hypoxic–ischemic encephalopathy. Brain MR angiography demonstrated cerebral vessel tortuosity together with distal branch narrowing in the middle cerebral artery, anterior cerebral artery, and posterior cerebral artery territories (Figure 4).

At the last follow-up, her weight was 8.5 kg (−1.89 SDS), her length was 84 cm (0.65 SDS), and her head circumference was 47.5 cm (0.19 SDS). She was able to hold her head upright, sit with support, and demonstrate ocular tracking; however, no clinically meaningful words were reported. Molecular analysis identified a homozygous nonsense variant in *SLC2A10* (NM_030777.4): c.485G > A/p.(Trp162Ter). The variant was classified as pathogenic according to the current ClinVar clinical significance displayed in VarSome. The clinical, vascular, and molecular findings were consistent with ATS.

### 2.3. Genetic Results

A novel homozygous nonsense variant (c.485G > A/p.(Trp162Ter)) was identified in the proband upon whole-exome sequencing analysis. To assess variant segregation within the family, molecular analysis of first-degree relatives was performed; the phenotypically unaffected sibling was found to harbor the variant in the heterozygous form, and both parents were verified to be heterozygous carriers. This segregation pattern is fully concordant with an autosomal recessive inheritance model.

**Figure 5:**
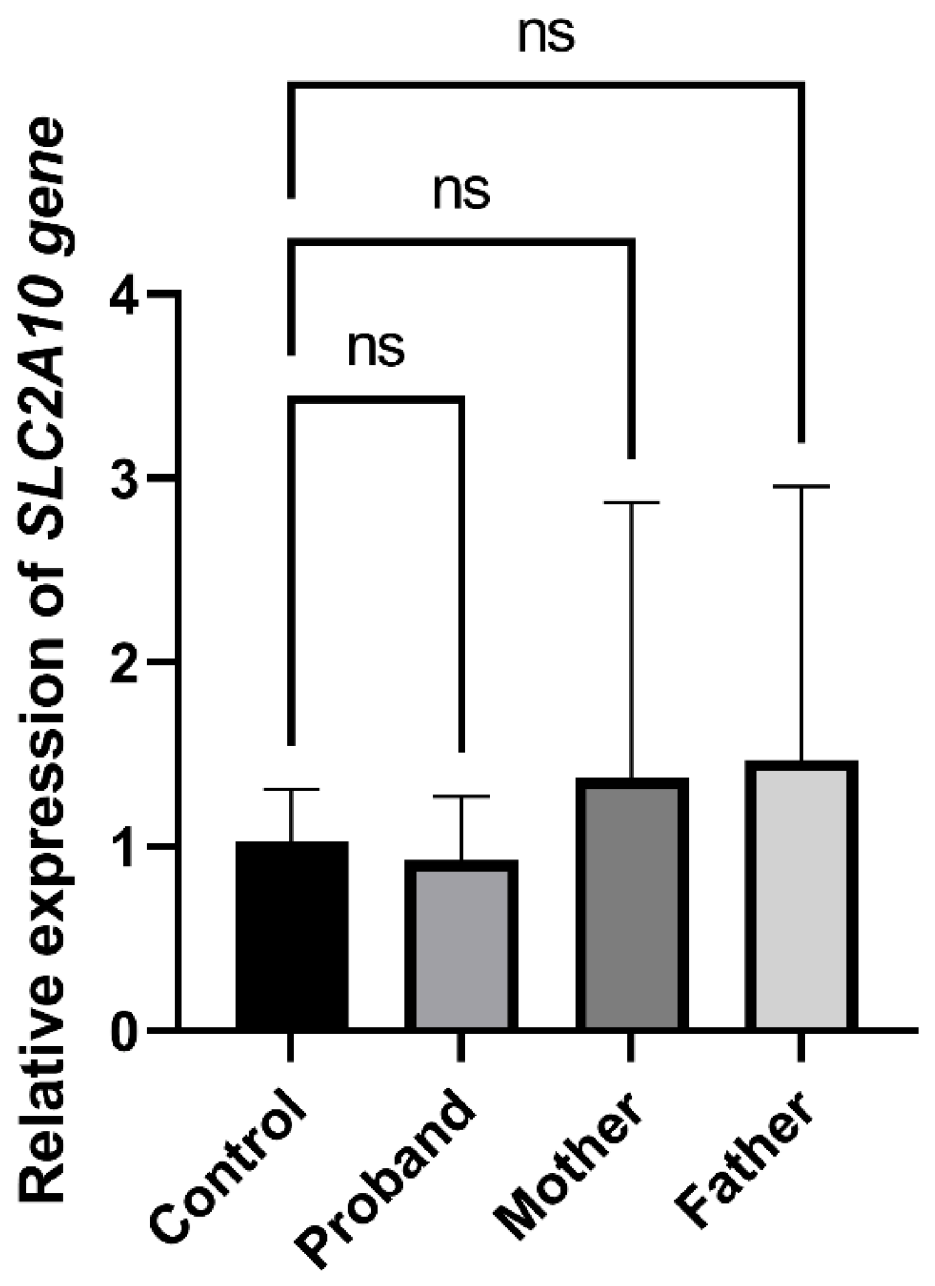
No significant change in *SLC2A10* gene expression was observed in the siblings compared to the control group (ns: not significant).

To assess the functional consequence of the identified variant at the mRNA level, relative *SLC2A10* gene expression was evaluated by qRT-PCR in peripheral blood samples obtained from the proband, both heterozygous carrier parents, and an unrelated healthy control. Primers were designed to flank the variant site, with the forward primer positioned upstream and the reverse primer downstream of the c.2836C > T variant, thereby generating an amplicon that spans the premature termination codon. Contrary to the anticipated reduction in transcript abundance, *SLC2A10* mRNA levels in the proband were not significantly different from those of the healthy control (ns: not significant). Similarly, no statistically significant differences in *SLC2A10* expression were detected between the control and either the heterozygous mother or father (*p* > 0.05 for all comparisons). Notably, both carrier parents exhibited a modest, non-significant trend toward elevated expression relative to the control (Figure 5).

### 2.4. Bioinformatic Results

#### 2.4.1. Protein Modeling and Druggability Site Prediction Results

**Figure 6:**
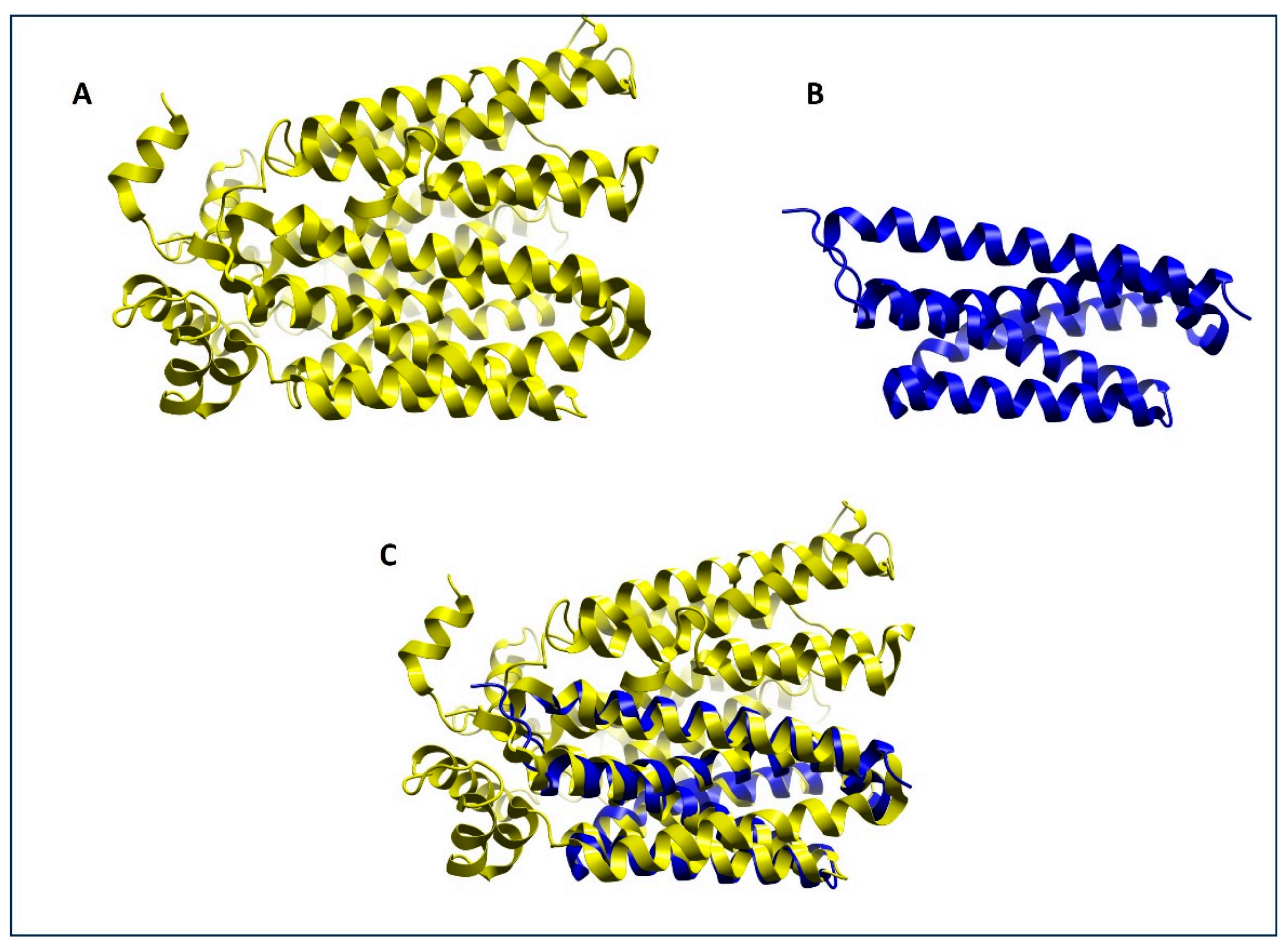
Structural modeling and alignment of the wild-type and mutant proteins. (**A**) Predicted three-dimensional structure of the wild-type protein (yellow). (**B**) Predicted three-dimensional structure of the mutant protein (blue). (**C**) Superimposition of the wild-type (yellow) and mutant (blue) models.

Structural modeling demonstrated that the variant resulted in a substantial loss of structural elements compared with the wild-type protein. The mutant model retained only a limited α-helical core, whereas several helices present in the wild-type structure were absent in the mutant. Structural superimposition confirmed that only a portion of the mutant protein aligned with the wild-type structure, indicating a pronounced alteration of the overall protein architecture (Figure 6).

**Figure 7:**
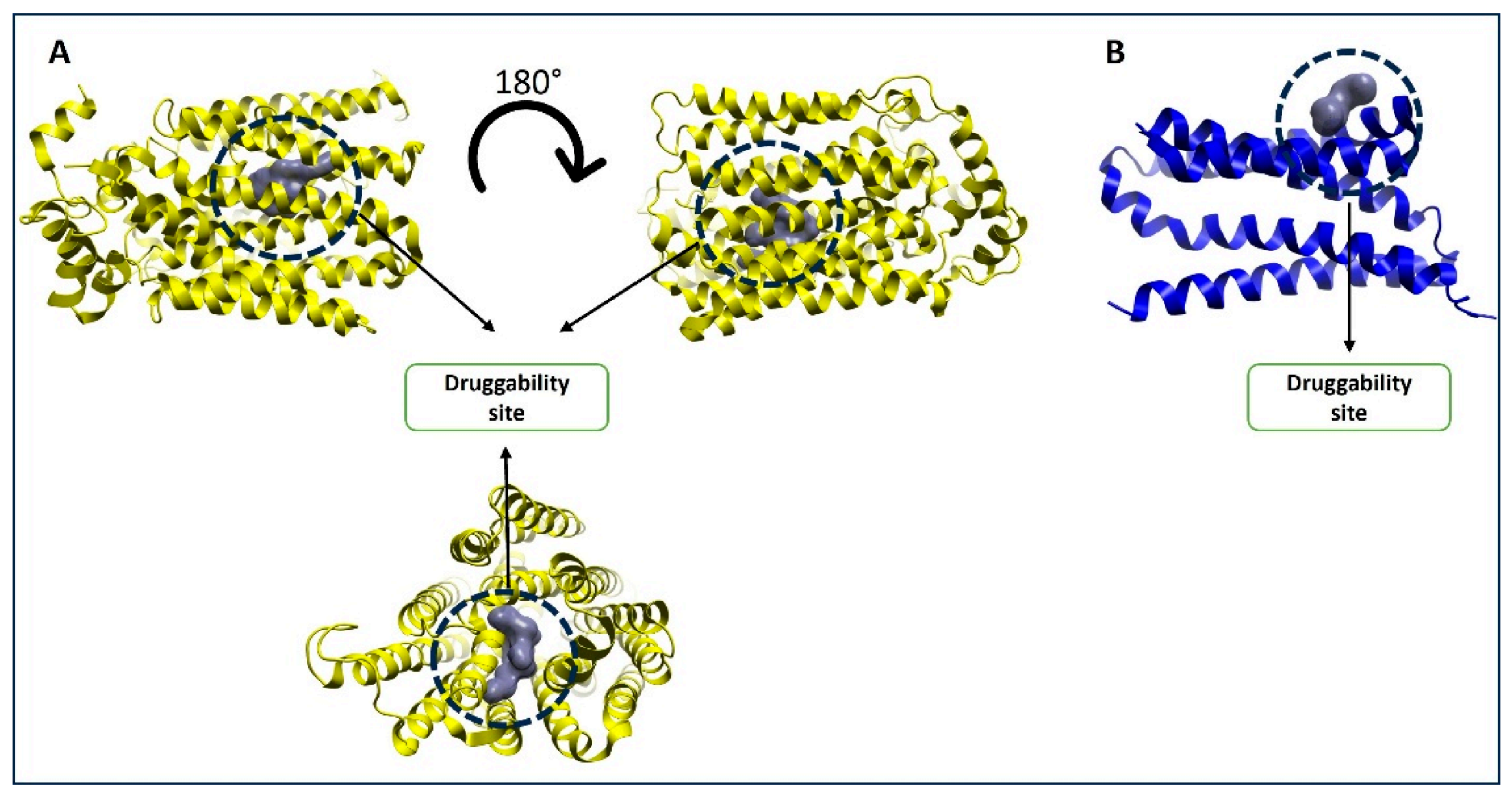
Druggability pocket prediction for the wild-type and mutant proteins. (**A**) Predicted druggable pocket of the wild-type protein (yellow), shown from two orientations related by a 180° rotation (top) and from a perpendicular view (bottom). (**B**) Predicted druggable pocket of the mutant protein (blue), shown in the corresponding orientation.

Druggability analysis revealed notable differences between the wild-type and mutant structures (Figure 7). In the wild-type protein, the predicted druggable pocket was located within a well-defined internal cavity surrounded by multiple α-helical segments, forming an enclosed-binding environment. The pocket occupied a relatively large and structurally supported region, suggesting favorable conditions for ligand accommodation and its stabilization.

In contrast, the mutant structure exhibited a druggable site localized within a considerably smaller framework. Owing to the loss of several surrounding secondary structural elements, the predicted pocket appeared more exposed and less deeply embedded within the protein. These structural alterations may influence pocket geometry, accessibility, and ligand-binding properties. The observed differences suggest that the variant could modify the physicochemical characteristics of the binding region and potentially affect the interaction between the protein and small molecules.

#### 2.4.2. Prediction of Transmembrane Helices Results

**Figure 8:**
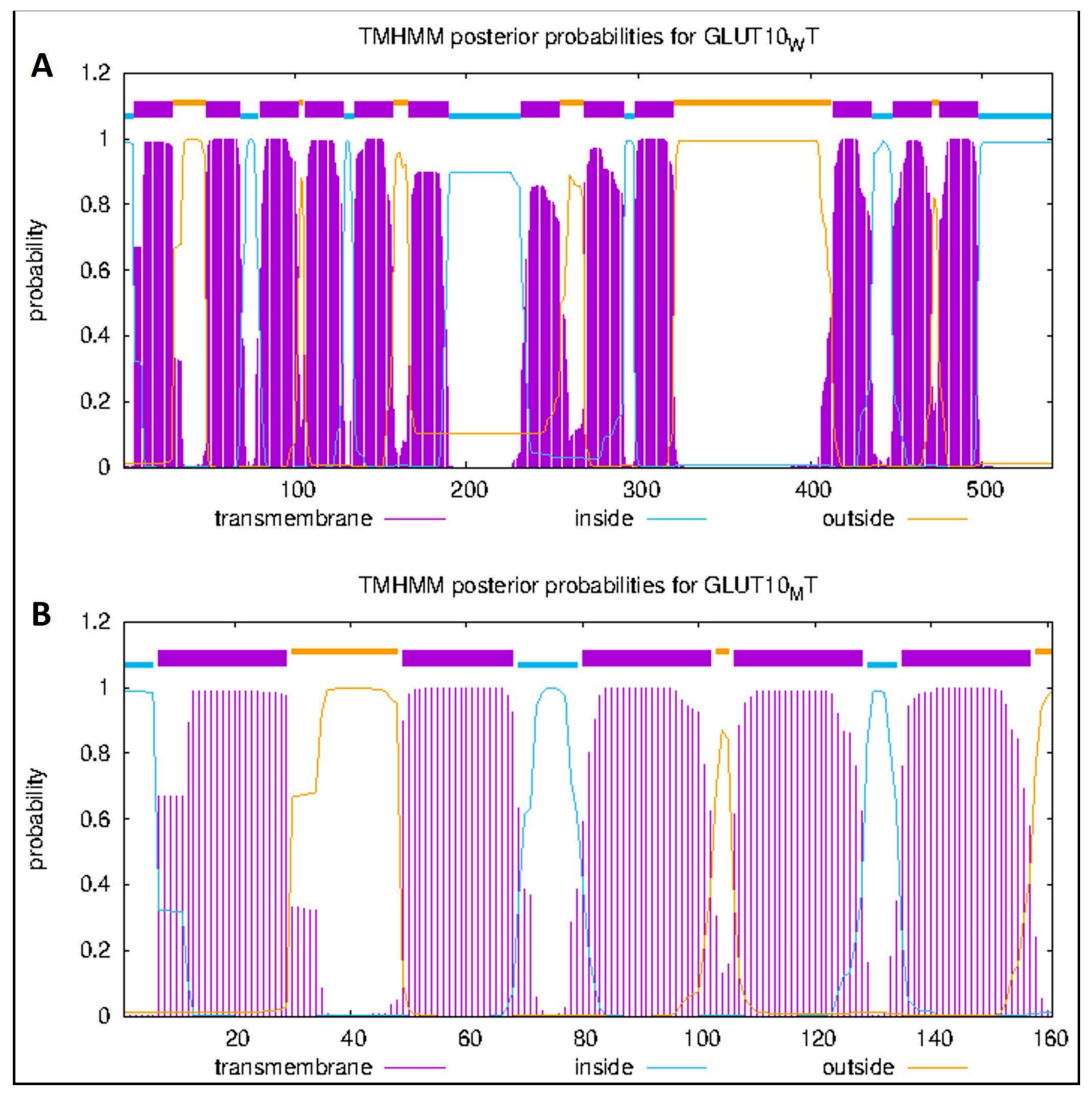
Transmembrane analysis of wild type (**A**) and mutant type (**B**).

TMHMM analysis demonstrated that wild-type GLUT10 contains 12 transmembrane helices. In contrast, the mutant protein showed a drastic reduction in the number of predicted transmembrane helices, with only five membrane-spanning segments remaining in the structure (Figure 8). This alteration resulted in a severely disrupted membrane topology and suggests a substantial impairment of the structural framework required for normal transporter function.

#### 2.4.3. Prediction Molecular Docking and MM/GBSA Calculation

All tested ligands exhibited more favorable binding energies against the wild-type structure than the mutant type (Table 2). Docking scores for the wild-type protein ranged from −7.469 to −5.772 kcal/mol, whereas the mutant protein displayed substantially weaker binding affinities, with docking scores ranging from −4.013 to −2.983 kcal/mol.

Among the investigated compounds, the compound with Pubchem ID 892 exhibited the strongest binding affinity towards the wild-type protein, with a docking score of −7.469 kcal/mol, followed by PubChem IDs 439213 (−7.394 kcal/mol) and 18950 (−7.305 kcal/mol). In contrast, the highest binding affinity observed for the mutant protein was recorded for PubChem ID 5984 (−4.013 kcal/mol), while the remaining compounds showed docking scores between −3.962 and −2.983 kcal/mol. However, docking that relies on simplified energy models is primitive. Therefore, we also performed MM/GBSA calculation to offer more realistic assessments of ligand binding affinity [17,18].

Overall, the mutant structure consistently demonstrated reduced ligand-binding affinities compared with the wild-type protein. The observed decrease in docking and MM/GBSA scores suggests that the variant alters the architecture of the binding pocket and may impair favorable protein–ligand interactions.

#### 2.4.4. Molecular Dynamics Results

To further evaluate the stability of the protein–ligand complexes, a 300 ns molecular dynamics simulation was performed for the complex formed between GLUT10 and the top two-ranked ligands shown in Table 2.

**Figure 9:**
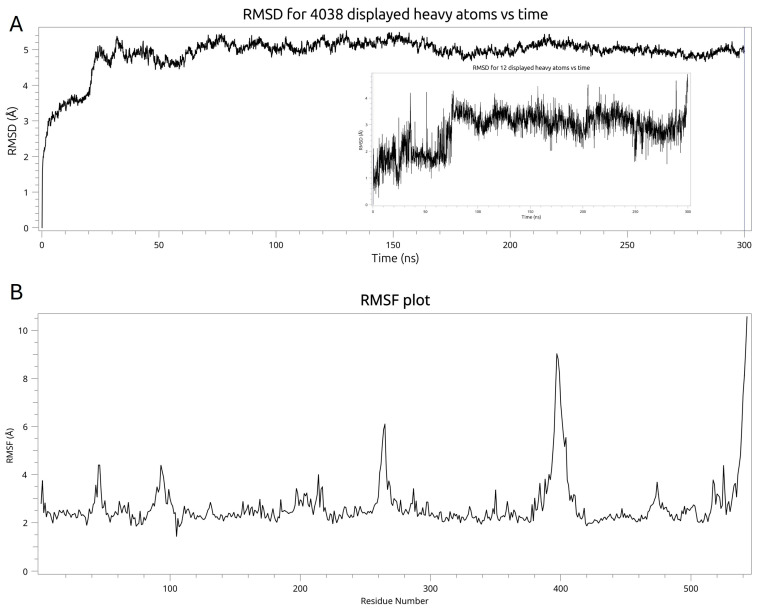
Molecular dynamics analysis of the wild-type protein in complex with PubChem CID 892. (**A**) RMSD profile of the protein backbone with ligand RMSD (inset). (**B**) RMSF plot of wild-type-ligand complex.

For the wild-type GLUT10/Pubchem ID 892 complex, the protein RMSD profile showed that the RMSD increased during the initial phase of the simulation and reached equilibrium after approximately 50 ns. Subsequently, the system remained relatively stable, at around 5 Å throughout the simulation time. Likewise, after an initial repositioning, the ligand stays at the binding pocket showing that the RMSD value fluctuated within a relatively constant range, stabilizing at ~3 Å, and suggesting that the ligand binds firmly to the binding pocket (Figure 9A). To get more insight into details of the fluctuation behaviors of each amino acid during the simulation, we also depicted the RMSF graph of protein. The RMSF analysis demonstrated that the complex fluctuated between 2–3 Å with two regions having high fluctuations (Figure 9B). Subsequent analysis of these two regions showed that the amino acids belong to loop regions that are a large distance from the binding site.

**Figure 10:**
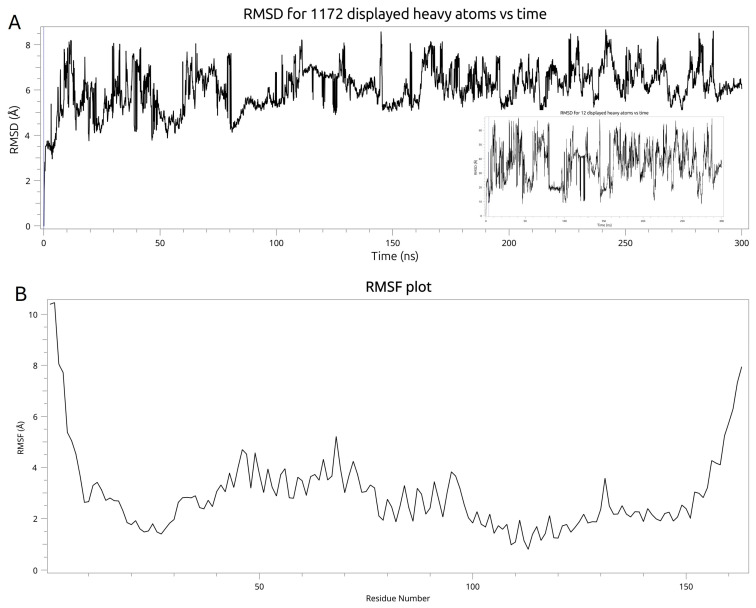
Molecular dynamics analysis of the mutant-type protein in complex with PubChem CID 892. (**A**) RMSD profile of the protein backbone with ligand RMSD (inset). (**B**) RMSF plot of mutant type-ligand complex.

On the other hand, the RMSD profile of mutant type GLUT10/Pubchem ID 892 complex exhibited substantially larger RMSD fluctuations during the entire simulation period. RMSD values varied between approximately 4 Å and 8.5 Å, suggesting unstable protein conformation. The ligand RMSD profile also displayed noticeable fluctuations during the simulation (Figure 10A). These large RMSD values indicate that the ligand does not stay in the binding pocket. Indeed, visual inspection of the simulation showed that the ligand diffuses away from the binding site directly after the beginning of the simulation. The RMSF analysis demonstrated that the majority of residues exhibited relatively low to moderate flexibility but were quite unstable compared with the wild-type protein (Figure 10B).

**Figure 11:**
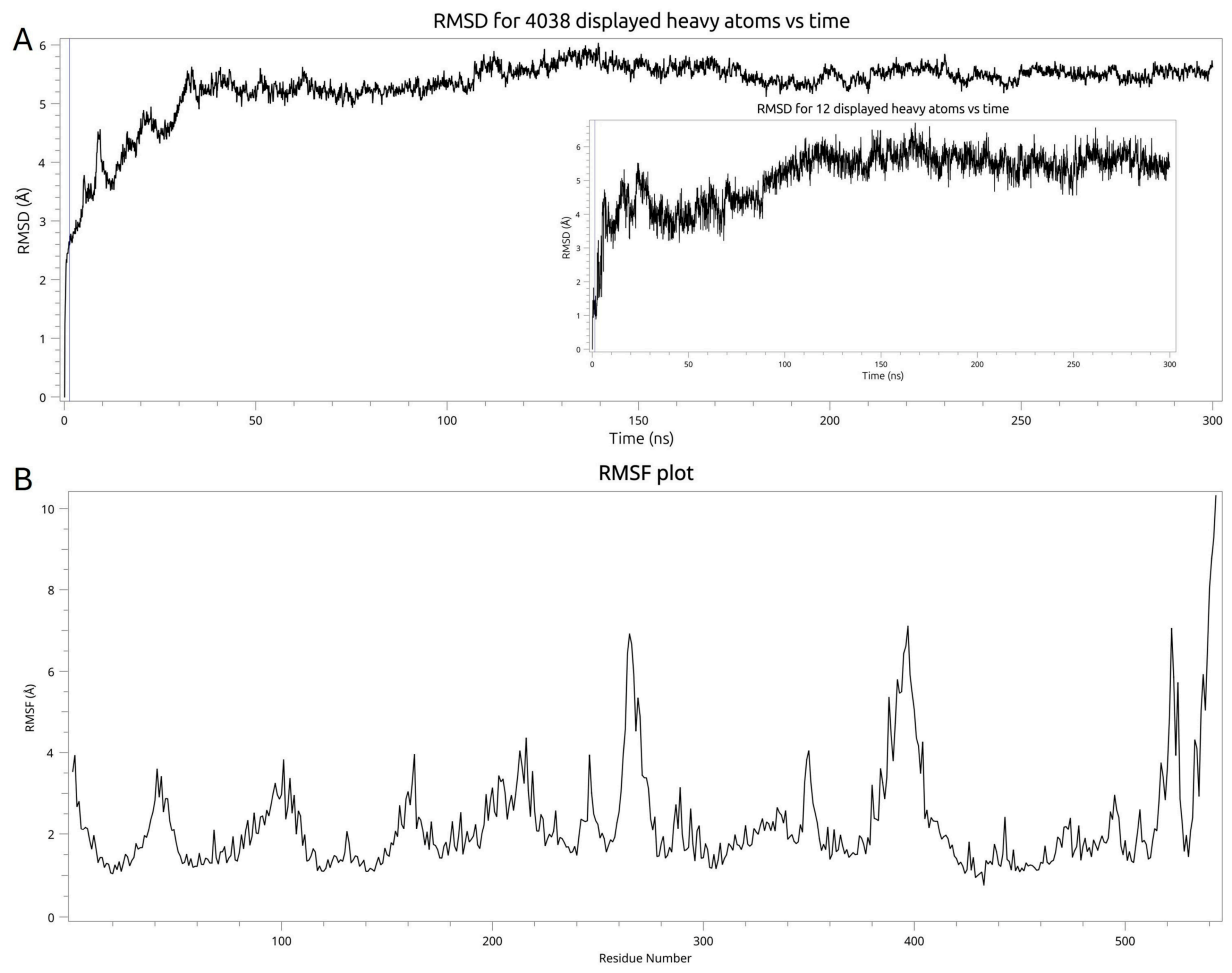
Molecular dynamics analysis of the wild-type protein in complex with PubChem CID 5984. (**A**) RMSD profile of the protein backbone with ligand RMSD (inset). (**B**) RMSF plot of wild-type-ligand complex.

For the wild-type GLUT10/Pubchem ID 5984 complex, the RMSD profile indicated that, the protein RMSD gradually increases to approximately 5.3 Å during the first 30–40 ns. Then, the RMSD fluctuates within a relatively narrow range (approximately 5.3–5.8 Å) throughout the simulation. The ligand RMSD profile exhibited a similar behavior suggesting stable binding of the ligand to the binding site (Figure 11A). The RMSF analysis further demonstrated that the majority of protein residues exhibited low flexibility, with RMSF values ranging between 1 Å and 3 Å (Figure 11B).

**Figure 12:**
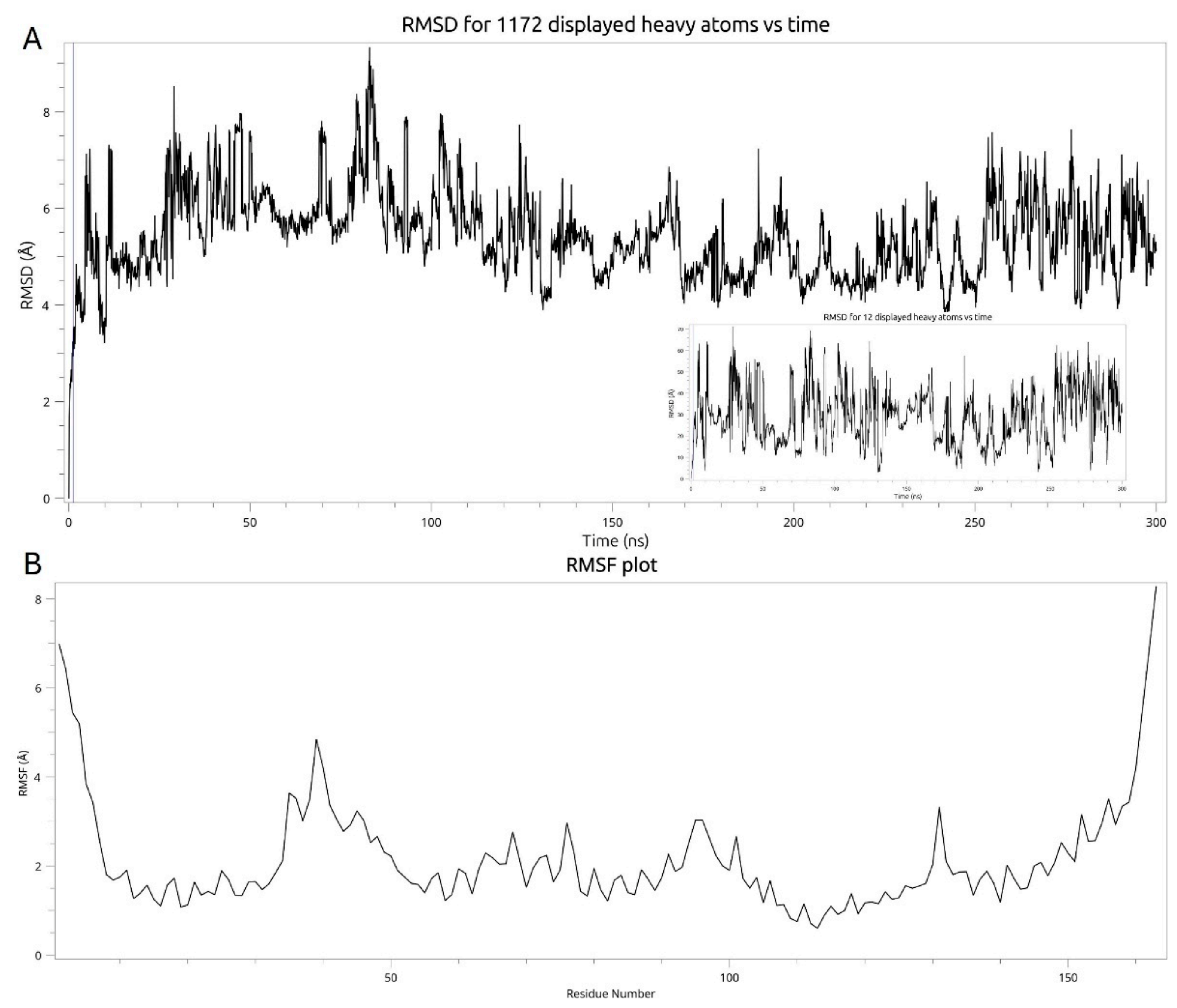
Molecular dynamics analysis of the mutant-type protein in complex with PubChem CID 5984. (**A**) RMSD profile of the protein backbone with ligand RMSD (inset). (**B**) RMSF plot of mutant type-ligand complex.

For the mutant type GLUT10/Pubchem ID 5984 complex, the RMSD profile showed that mutant form underwent greater conformational flexibility than the wild-type complex. Similarly, ligand RMSD displayed higher fluctuation throughout the simulation (Figure 12A). The RMSF analysis indicated that the majority of the residues fluctuated in many regions during the simulation, (Figure 12B). This also confirms that the mutant type is structurally less stable than the wild type.

#### 2.4.5. Protein–Ligand Interaction Results

**Figure 13:**
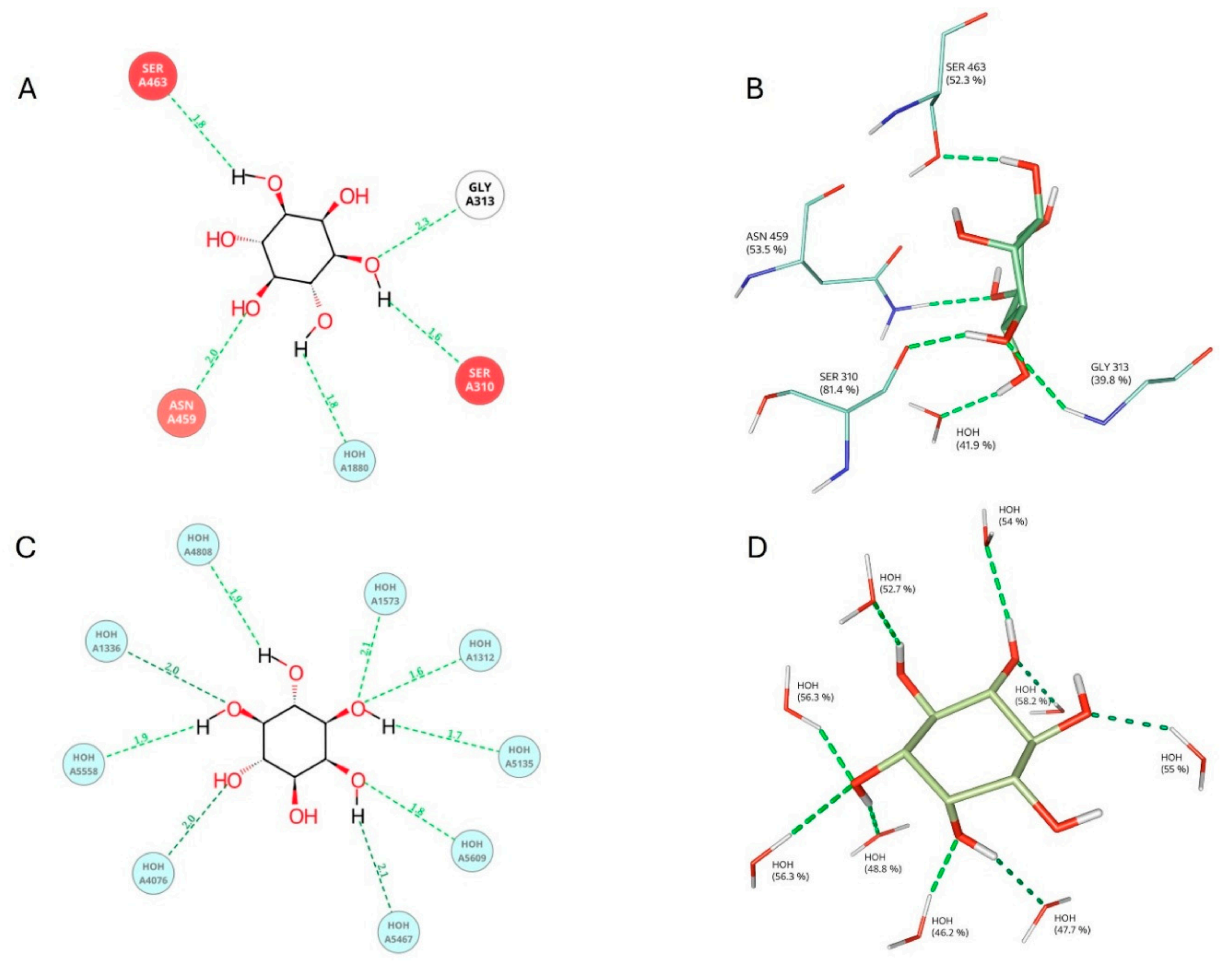
Protein–ligand interaction analysis of PubChem CID 892. (**A**) Two-dimensional interaction map of the wild-type complex. (**B**) Three-dimensional interaction profile of the wild-type complex. (**C**) Two-dimensional interaction map of the mutant complex. (**D**) Three-dimensional interaction profile of the mutant complex. Hydrogen bonds were represented as green dashed lines.

Protein–ligand interactions were analyzed using the representative structure of the most populated cluster. In the wild-type GLUT10/Pubchem ID 892, established hydrogen bonds with Ser310, Gly313, Ser463, and Asn459, together with a water-mediated hydrogen bond (Figure 13A,B). In contrast, the mutant GLUT10/Pubchem ID 892 complex exhibited no interaction with any amino acid residues in the binding pocket. The only interactions observed are with the water molecules present in the binding region. (Figure 13C,D). This clearly explains why the ligand does not stay in the binding site.

**Figure 14:**
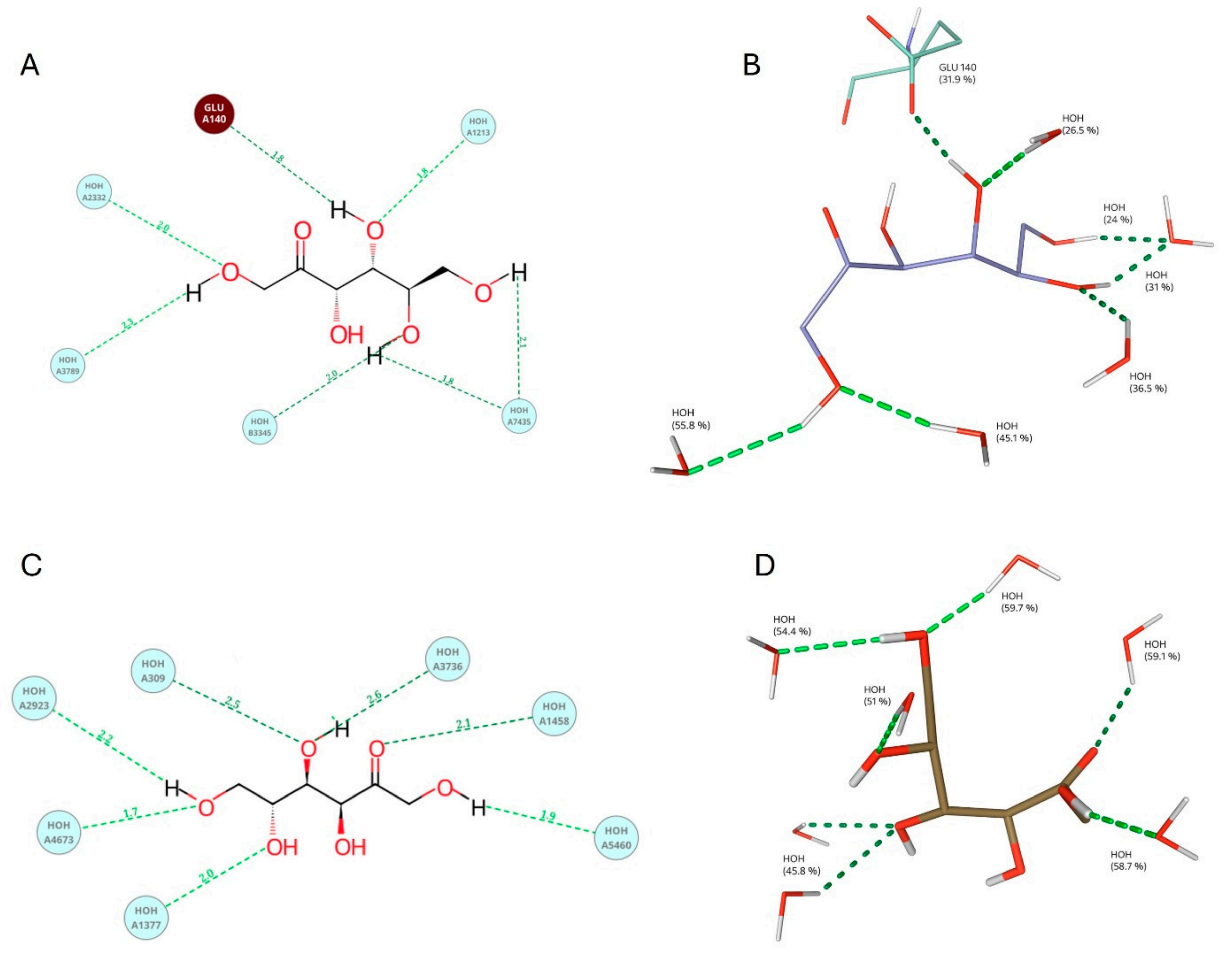
Protein–ligand interaction analysis of PubChem CID 5984. (**A**) Two-dimensional interaction map of the wild-type complex. (**B**) Three-dimensional interaction profile of the wild-type complex. (**C**) Two-dimensional interaction map of the mutant complex. (**D**) Three-dimensional interaction profile of the mutant complex. Hydrogen bonds were represented as green dashed lines.

In the wild-type GLUT10/Pubchem ID 5984 complex, the ligand established a direct hydrogen bond with Glu140 together with water-mediated hydrogen bonds (Figure 14A,B). On the contrary, the mutant GLUT10/Pubchem ID 5984 complex exhibited different interaction patterns including a hydrogen bond with water-mediated hydrogen bonds (Figure 14C,D).

## 3. Discussion

It is known that the variant occurred in the *SLC2A10* gene; encoding glucose transporter GLUT10 protein causes the arterial tortuosity syndrome (ATS) [1]. In this study, nine reported ligands were used for the in silico analysis. The bioinformatics result indicated that the two ligands with Pubchem ID 892 and 5984 are bound to the wild-type GLUT10 with more favorable energy than the mutant type (Table 2). The ligands with 892 and 5984 IDs are known as myoinositol and D-fructose, respectively [19].

A qRT-PCR analysis was performed using RNA samples obtained from peripheral blood to assess the functional impact of the homozygous nonsense variant (c.485G > A/p.(Trp162Ter)) identified in the *SLC2A10* gene. No statistically significant difference in *SLC2A10* mRNA expression was detected between the proband, heterozygous carrier parents, and healthy controls (Figure 5). Tissue selection is of critical importance in the interpretation of this finding. When the GTEx database is used as a reference (https://gtexportal.org/home/gene/SLC2A10, date of access: 5 March 2026), it can be seen that the basal expression level of *SLC2A10* in the peripheral blood is virtually zero. The reliability of RNA analysis is severely limited in genes with low TPM (transcripts per million) values in peripheral blood; this situation can lead to significant interpretative difficulties, particularly in studies aimed at evaluating variant regions. Therefore, it is likely that the finding of ‘no significant difference’ reflects the low basal expression in this tissue rather than nonsense-mediated mRNA decay (NMD) escape. Indeed, as the expression level is negligible, qRT-PCR measurements become susceptible to stochastic sampling error, thereby hindering the interpretation of the data obtained [20]. As a prerequisite, it requires the presence of sufficient basal expression in the tissue being analyzed. Given the complexity and variability of the NMD mechanism, determining NMD status using qPCR alone is insufficient; the use of complementary methods, including validation at the protein level, is recommended [21,22]. Consequently, it is considered that advanced gene expression analyses and validation studies at the protein level, supported by the application of an NMD inhibitor in suitable cell models (such as patient dermal fibroblasts in which SLC2A10 is physiologically active) will provide a more reliable understanding of the molecular effects of this variant.

ATS is traditionally described as a systemic arteriopathy characterized by elongation, tortuosity, stenosis, and aneurysmal remodeling of large- and medium-sized arteries. The present case, involving a patient with a homozygous *SLC2A10* nonsense variant, c.485G > A/p.(Trp162Ter), contributes additional detail to the expanding clinical spectrum of genetically confirmed ATS, with particular relevance to intracranial vascular involvement. Alongside several well-recognized extracranial features—such as dysmorphic facial characteristics, increased skin elasticity, joint laxity, hiatal hernia, bilateral inguinal hernias, congenital cardiac findings, and prominent tortuosity of the aortic arch and its major branches—the patient also demonstrated clinically relevant neurovascular abnormalities. Notably, early neurodevelopment was reported as age-appropriate, and neurological deterioration became evident only after surgical repair of a hiatal hernia. In the postoperative period, neuroimaging revealed diffusion restriction compatible with hypoxic–ischemic encephalopathy, together with MR angiographic evidence of cerebral vessel tortuosity and distal branch narrowing within the MCA, ACA, and PCA territories. While intracranial arterial tortuosity was described previously in ATS, its association here with clinically significant neurological injury places this case among a smaller group of patients in whom cerebrovascular involvement appears to have more than purely radiological significance. The review of the genetically characterized cases summarized in Table 1 underscores the considerable heterogeneity of the neurovascular findings in ATS. In a number of reported patients, pronounced intracranial or cervical arterial tortuosity was identified in the absence of documented neurological events [1,4,6,7,11,15,16]. Conversely, overt neurological complications have also been reported, including hemiplegia related to internal carotid artery dissection, ischemic stroke with persistent deficits, neonatal intraventricular or parenchymal hemorrhage accompanied by diffuse ischemic injury, recurrent transient ischemic attacks, and intracranial aneurysm formation [5,8,11,12]. Taken together, these observations suggest that neurological expression in ATS spans a wide spectrum and that the presence or absence of clinical symptoms does not reliably predict the extent of intracranial vascular involvement. The present case is of particular interest because the neurological insult occurred in the perioperative setting rather than as an unprovoked cerebrovascular event. We do not interpret this temporal association as indicating a direct causal relationship between ATS and hypoxic–ischemic injury. However, the coexistence of diffuse systemic arteriopathy, cerebral arterial tortuosity, and distal intracranial branch narrowing raises the possibility that cerebrovascular reserve may be reduced in some individuals with ATS. In such a context, perioperative physiological stressors could potentially contribute to neurological vulnerability. Similar patterns of carotid or vertebrobasilar involvement, intracranial narrowing, aneurysm formation, and ischemic manifestations have been variably described across different ATS genotypes [5,11,12,14,15]. From a clinical perspective, these observations support heightened awareness of possible cerebrovascular involvement in ATS, particularly in patients undergoing major surgical procedures. Although robust evidence is lacking, careful perioperative hemodynamic management and consideration of neuroimaging may be prudent in individuals with extensive arterial disease. With respect to genotype, the cases summarized in Table 1 indicate that both truncating and missense *SLC2A10* variants have been associated with intracranial vascular abnormalities or clinically apparent neurovascular events. Several recurrent loss-of-function variants—including c.510G > A/p.(Trp170Ter), c.685C > T/p.(Arg229Ter), and c.1334delG/p.(Gly445GlufsTer40)—have been reported in multiple patients displaying a broad range of neurological outcomes, from asymptomatic cerebrovascular tortuosity to stroke or TIA-like episodes [1,5,6,11,12,13,15]. The c.485G > A/p.(Trp162Ter) variant identified in our patient likewise introduces a premature termination codon and is currently classified as pathogenic in ClinVar and VarSome. Although this variant was previously submitted to ClinVar, the present report appears to provide the first detailed description of the clinical and neurovascular phenotype associated with homozygosity for this variant [23]. Despite these observations, available data do not support a simple or predictable genotype–phenotype correlation in ATS. Individuals carrying identical variants may exhibit markedly different degrees of systemic, pulmonary, skeletal, ocular, and neurovascular involvement, while some missense variants are associated with severe arterial phenotypes. This variability emphasizes the need for cautious interpretation of individual variants, irrespective of their molecular classification. An additional consideration is the variable concordance between clinical observations and public database classifications. In reviewing the variants included in Table 1, we observed that several variants reported in clinically well-characterized ATS cases currently have conflicting or absent ClinVar classifications, whereas others are consistently classified as pathogenic or likely pathogenic. This discrepancy is particularly relevant for rare missense variants and for conditions in which phenotypic evidence may exceed available population-level data. Accordingly, variant interpretation in ATS should remain primarily phenotype-driven, with database annotations serving as supportive rather than determinative evidence. The extracranial features observed in our patient are also consistent with the established ATS phenotype. Hiatal and inguinal hernias, joint laxity, increased skin elasticity, craniofacial dysmorphism, congenital cardiac anomalies, pulmonary stenosis, and extensive arterial tortuosity have all been repeatedly documented in previous reports [4,5,6,7,9,10,11,15]. The presence of consanguinity and a homozygous truncating variant further supports the diagnosis in keeping with the autosomal recessive inheritance pattern of ATS. The family history of congenital heart disease, including a sibling who died with tetralogy of Fallot, is noteworthy, although definitive interpretation is limited in the absence of molecular confirmation.

In conclusion, this case reinforces several clinically relevant considerations. First, cerebral and cervical vascular imaging should be considered as part of the phenotypic evaluation in genetically confirmed ATS, even in the absence of overt neurological symptoms. Second, perioperative neurological deterioration in ATS warrants careful assessment of both parenchymal injury and intracranial arterial morphology. Third, the combination of a pathogenic *SLC2A10* loss-of-function variant with systemic arterial tortuosity, connective tissue manifestations, hernias, and intracranial vascular abnormalities provides strong support for the diagnosis in this patient. The principal limitation remains the predominance of case reports and small series in the literature, with heterogeneous imaging approaches and limited longitudinal follow-up. Future genotype-informed studies incorporating standardized vascular imaging may help clarify whether specific intracranial arterial patterns confer increased neurological risk in ATS.

## 4. Materials and Methods

### 4.1. Literature Search, Case Selection and Ethics

A targeted PubMed based literature search was performed to identify previously reported arterial tortuosity syndrome (ATS) cases with documented *SLC2A10* variants and neurological, neurovascular, or intracranial/cerebrovascular involvement. During the literature screening process, one additional article that was not indexed in PubMed was also included because it fulfilled the predefined inclusion criteria and provided patient level *SLC2A10* genotype and relevant neurovascular data. Publications were screened at the title/abstract and full-text levels. Cases were included when patient level genotype data and relevant neurological or cerebrovascular findings were available.

The study protocol received ethical approval from the Clinical Research Ethics Committee of Biruni University (Reference No: 2024-BİAEK/17-43, dated 26 January 2026). Written informed consent was duly obtained from each participant and/or their legally designated guardians prior to any study-related procedures. The research was conducted in strict accordance with the ethical principles set forth in the Declaration of Helsinki by the World Medical Association.

### 4.2. Genetic Analysis

Peripheral blood samples were collected from the participant, and genomic DNA extraction was carried out using a commercially available kit in strict accordance with the manufacturer’s protocol (The PureLink Genomic DNA Mini Kit, Thermo Fisher Scientific, Waltham, MA, USA). The purity and concentration of the isolated DNA were evaluated by spectrophotometric analysis (Thermo Scientific NanoDrop, Waltham, MA, USA), and samples were stored under conditions suitable for long-term preservation until downstream applications.

Whole exome sequencing (WES) was employed to comprehensively interrogate coding regions of the genome. Raw sequencing reads were mapped to the GRCh37/hg19 human reference genome assembly, and a standardized bioinformatics pipeline was applied for variant calling and quality-based filtering. Variants of potential clinical relevance were shortlisted by integrating allele frequency data from publicly available population databases, prior literature reports, and computational pathogenicity predictions derived from multiple, in silico tools. Final variant classification followed the framework outlined in the 2015 ACMG/AMP guidelines for the interpretation of sequence variants [24].

To elucidate the functional impact of the identified variant on gene expression, quantitative real-time PCR (qPCR) was performed. Total RNA was isolated from peripheral blood and subsequently converted to complementary DNA (cDNA) through reverse transcription. Each amplification reaction was assembled in a total volume of 20 µL, comprising cDNA template, a commercially available 2x qPCR master mix, and gene-specific primer pairs targeting *SLC2A10* (Forward 5′ → 3′: TGCCCTCAACTATGCACTGG; Reverse 5′ → 3′: TGTACCAGCAGGGAGGAAGA). Thermal cycling was initiated with a denaturation phase, followed by iterative cycles of denaturation, primer annealing, and extension under empirically optimized temperature conditions. Expression levels were quantified by applying the 2^−^ΔΔCt method, with a housekeeping gene serving as the endogenous reference for normalization (*GAPDH* Forward 5′ → 3′: GAAGGTGAAGGTCGGAGTCA; Reverse 5′ → 3′: TGACAAGCTTCCCGTTCTCA). To confirm analytical reproducibility, all reactions were conducted in technical replicates.

### 4.3. In Silico Analysis

#### 4.3.1. Protein Modeling and Druggability Site Prediction

3D structures of wild-type and mutant type protein were predicted using the I-TASSER (https://aideepmed.com/I-TASSER/, access on 15 May 2026) server [25]. Structural refinement and energetic optimization were performed using GalaxyRefine (https://galaxy.seoklab.org/cgi-bin/submit.cgi?type=REFINE, access on 23 May 2026). The druggability site for both wild and mutant type was predicted by Cresset Flare Pro+ software (https://cresset-group.com/software/flare/).

#### 4.3.2. Prediction of Transmembrane Helices

The TMHMM server (https://services.healthtech.dtu.dk/services/TMHMM-2.0/, access on 25 May 2026) was used to predict the transmembrane helices for wild and mutant type of GLUT10.

#### 4.3.3. Molecular Docking

Nine reported potential in vitro substrates of GLUT10 were selected based on an extensive literature review identifying compounds previously reported to interact with GLUT10. Their 3D structures were retrieved from PubChem database (https://pubchem.ncbi.nlm.nih.gov/, access on 20 May 2026) and docked into the predicted druggability site for both wild- and mutant type. Then, the docking scores for each ligand were calculated and compared. Also, the interacted residues between ligand and wild and mutant types were indicated.

#### 4.3.4. Molecular Dynamics

Molecular dynamics simulations were conducted on the docking complex using Cresset’s Flare Pro+ software (Version 11.0.0) to elucidate intermolecular interactions and dynamic behavior under physiological conditions [26,27,28]. Protein and ligand atoms were parameterized using the AMBER force field, and the system was solvated using the transferable intermolecular potential 3P (TIP3P) water model, with the solvent box as orthogonal [29]. Then, for the neutralization of the system’s overall charge, the solvent ionic strength was adjusted to 0.15 M. Following system preparation and equilibration, the simulation was performed for 300 ns. Subsequently, the stability of the protein–ligand complex was assessed by analyzing the root mean-square deviation (RMSD) of heavy atoms in the protein and the root mean-square fluctuation (RMSF) of amino acids throughout the simulation.

#### 4.3.5. Protein–Ligand Interaction

Following the 300 ns molecular dynamics simulations, clustering analysis was performed to identify the dominant conformational states of each protein–ligand complex. The representative structure of the most populated cluster (highest population fraction) was selected for each system. Protein–ligand interactions, including hydrogen bonds, electrostatic interactions, aromatic (π) interactions, and water-mediated contacts, were subsequently analyzed using these representative conformations to identify the key interactions responsible for ligand stabilization within the binding pocket.

## Figures and Tables

**Table 1:** Reported *SLC2A10* variants and neurological/neurovascular findings in previously published arterial tortuosity syndrome (ATS) cases.

PN	Sex/Age	Neurological/Clinical Neurovascular Manifestations	Cardiovascular/Systemic Vascular Findings	Intracranial/Cerebrovascular Imaging Findings	Additional ATS-Related Findings	*SLC2A10* (NM_030777.4) Variant Nomenclature (cDNA/Protein)	Variant Type	Zygosity	CS	Reference
P1	F/Infant, exact age NR	Cerebral arterial tortuosity. Clinical neurological symptoms NR.	Generalized arterial tortuosity. Detailed systemic vascular findings for this individual NR.	MRA showed typical tortuosity of the cerebral arteries.	Typical facial phenotype including micrognathia, elongated face, down-slanting palpebral fissures, blepharophimosis, and beaked nose.	c.510G > A/p.(Trp170Ter)	Nonsense	HM	P	Coucke et al. 2006 [1] (Family 1, IV:4)
P2	M/14 years	Hypotonia. Clinical cerebrovascular event NR.	Stenosis and tortuosity of the left pulmonary artery. Tortuosity of carotid and vertebral arteries.	Intracerebral artery tortuosity.	Typical facial features, keratoconus, obesity, and joint laxity.	c.1309G > A/p.(Glu437Lys), c.1330C > T/p.(Arg444Ter)	Missense + nonsense	CH	P/LP, P/LP	Drera et al. 2007 [4]
P3	M/8 months	Right-sided hemiplegia.	Generalized arterial tortuosity. Aberrant origin of aortic side branches reported in this patient.	Vascular imaging showed internal left carotid artery dissection.	Wrinkling of the skin of hands and feet.	c.425G > T/p.(Gly142Val)	Missense	HM	ClinVar classification NR	Callewaert et al. 2008 [5] (Patient J:II-1)
P4	M/23 years	Stroke with long-term neurological disability. Sudden right eye pain followed by diplopia.	Generalized arterial tortuosity. Aortic root dilation. Long stenotic stretch extending into the descending aorta. Focal stenosis of the left carotid artery. Borderline main pulmonary artery dilation.	Neurological workup confirmed marked cerebral arterial tortuosity.	Cutis laxa, recurrent inguinal hernias, macrocephaly, tracheomegaly, and duplication of the right collecting system.	c.1276G > T/p.(Gly426Trp), c.1334delG/p.(Gly445GlufsTer40)	Missense + frameshift	CH	P/LP, P	Callewaert et al. 2008 [5] (Patient B:II-2)
P5	F/27 years	Clinical cerebrovascular event NR. Low-dose acetylsalicylic acid was started postpartum for empiric stroke prophylaxis due to cervical and intracranial arterial tortuosity.	Multiple pulmonary artery aneurysms and diffuse pulmonary arterial tortuosity. Dilatation of the right pulmonary artery. Mildly tortuous and elongated aortic arch without frank coarctation. Dilated and tortuous carotid arteries and marked tortuosity of vertebral arteries. Mild mitral and tricuspid regurgitation with biatrial enlargement.	Head and neck MRI showed marked tortuosity of the intracranial vasculature. Circle of Willis MRI showed an elongated tortuous right carotid siphon with tortuous middle cerebral artery, together with tortuous cervical internal carotid and vertebral arteries.	Long narrow face, sagging cheeks, down-slanting palpebral fissures, beaked nose, micrognathia, dough-like skin, joint laxity, rectal prolapse, inguinal hernia, hiatal hernia, recurrent infections including pneumonia, and Chilaiditi syndrome. Successful cesarean delivery with intensive perioperative monitoring.	c.685C > T/p.(Arg229Ter)	Nonsense/truncating	HM	P	Allen et al. 2009 [6]
P6	M/2 years	Clinical cerebrovascular event NR.	Annuloaortic ectasia. Tortuosity of the aortic arch. Tortuosity and elongation of the descending and abdominal aorta and other large- and medium-sized arteries.	MRA showed tortuosity and elongation of large- and medium-sized cerebral arteries.	Early-onset pulmonary emphysema, recurrent respiratory infections, macrosomia/overgrowth at birth, pectus excavatum, inguinal hernia, Morgagni foramen hernia, sagging cheeks, and hyperextensible skin. Joint and ocular involvement were not observed.	c.417T > A/p.(Tyr139Ter), c.692G > A/p.(Arg231Gln)	Nonsense + missense	CH	P/LP, P	Takahashi et al. 2013 [7]
P7	M/8 years	Clinical cerebrovascular event NR.	Severe bilateral pulmonary artery stenosis. Pulmonary hypertension. Vascular tortuosity.	Intracranial aneurysm.	Joint hypermobility, skin hyperelasticity, myopia, thin corneas, progressive myopia and astigmatism, keratoglobus, and deep stromal corneal opacities.	c.394C > T/p.(Arg132Trp), c.800delC/p.(Ser268GlnfsTer12)	Missense + frameshift	CH	P/LP, P	Hardin et al. 2018 [8] (Case 1)
P8	F/10 years	Migraine.	Marked tortuosity of carotid and vertebral arteries. Tortuosity of aortic arch branches. Pulmonary artery tortuosity was not detected.	MRA/CT angiography showed marked tortuosity of cerebral arteries.	Congenital diaphragmatic hernia, generalized joint hypermobility, high palate, dental crowding, flexible flat feet, elongated face, beaked nose, and long philtrum.	c.254T > C/p.(Leu85Pro)	Missense	HM	Conflicting interpretations of pathogenicity	Kocova et al. 2018 [9] (Patient 1)
P9	M/2 months	Hypotonia. Clinical cerebrovascular event NR.	Severe hypertension. Left renal artery stenosis. Tortuous thoracic and abdominal aorta. Abnormalities of the pulmonary artery trunk and branches. Multiple arterial dilations. Ascending aorta dilation. Increased left ventricular mass.	Computed tomography demonstrated major tortuosity of the cerebral and cervical arteries.	Hypertrophic pyloric stenosis, arachnodactyly, cutis laxa, and excessive skin wrinkling.	c.737G > A/p.(Gly246Glu)	Missense	HM	ClinVar classification NR	Marcellus et al. 2018 [10]
P10	F/3 months	Stroke resulting in left hemiparesis.	Carotid arterial tortuosity. Aortic tortuosity, aortic root aneurysm, pulmonary artery stenosis, aortic stenosis, and other stenoses were not reported as present.	Cerebral arterial tortuosity.	Dyspnea at initial presentation. Broad forehead/frontal bossing, epicanthal folds, down-slanting palpebral fissures, beaked nose, micrognathia, myopia, velvety skin texture, hyperextensible skin, cutis laxa, pectus deformity, scoliosis, joint laxity, muscular hypotonia, emphysema, inguinal hernia, umbilical hernia, and hiatal hernia.	c.510G > A/p.(Trp170Ter)	Nonsense	HM	P	Beyens et al. 2018 [11] (Individual F2)
P11	F/Neonatal	Neonatal grade II intraventricular and parenchymal hemorrhage with diffuse ischemic brain changes.	No aortic tortuosity, pulmonary artery stenosis, aortic stenosis, or other stenoses were reported. Internal carotid and basilar arterial involvement was reported.	Internal carotid and basilar arterial involvement. Brain hemorrhage with diffuse ischemic changes.	Respiratory distress syndrome at initial presentation. Beaked nose, sagging cheeks, hyperextensible skin, urogenital abnormality, and dilated pyelocaliceal system.	c.243C > G/p.(Ser81Arg)	Missense	HM	P/LP	Beyens et al. 2018 [11] (Individual F3)
P12	M/21 years	Clinical cerebrovascular event NR.	Aortic tortuosity. Tortuosity of pulmonary, carotid, abdominal, and iliac arteries. Ventricular dilatation and ventricular hypertrophy. Pulmonary artery stenosis, aortic stenosis, other stenoses, and arterial aneurysms were not reported.	Brain MRI showed generalized tortuosity of the intracranial arteries.	Cardiomyopathy at initial presentation. Overgrowth/macrosomia at birth, long face, down-slanting palpebral fissures, beaked nose, long philtrum, high-arched palate, micrognathia, sagging cheeks, keratoconus, cutis laxa, pectus deformity, arachnodactyly, joint laxity, bronchial asthma, inguinal hernia, umbilical hernia, eventration of the left diaphragm, hiatal hernia, and right-eye amblyopia.	c.1A > G/p.(Met1?)	Start-loss/initiation codon variant	HM	Conflicting interpretations of pathogenicity	Beyens et al. 2018 [11] (Individual F29)
P13	M/2 months	Clinical cerebrovascular event NR.	Aortic tortuosity. Pulmonary arterial tortuosity. Aortic root aneurysm requiring surgery. Abnormal implantation of aortic branches. Other vascular stenosis involving the inferior vena cava.	Brain arterial tortuosity.	Term birth, hoarse voice, long face, epicanthal folds, beaked nose, malar hypoplasia, micrognathia, sagging cheeks, large ears, velvety skin texture, thin and hyperextensible skin, pectus deformity, arachnodactyly, joint laxity, and inguinal hernia.	c.727C > A/p.(Gln243Lys)	Missense	HM	LP	Beyens et al. 2018 [11] (Individual F38)
P14	M/33 years	Recurrent transient ischemic attacks characterized by expressive aphasia and right hemiparesis. Treated with intravenous recombinant tissue plasminogen activator with complete recovery.	Marked tortuosity, dilatation, and elongation of the main arteries. Increased carotid bifurcation intima-media thickness. Aortic sinus dilatation with moderate aortic valve incompetence. Previous valve-sparing ascending aortic replacement. Thoracic and abdominal vascular tortuosity without stenosis. Moderate left ventricular hypertrophy with diastolic dysfunction.	Brain CT showed no hyperacute ischemic signs. Brain MRI showed no acute ischemic lesions. Intracranial and supra-aortic MRA showed marked vessel tortuosity without dissection, aneurysmal lesions, or hemodynamically significant stenoses.	Cardiac arrest at delivery without brain damage, normal psychophysical development, vomiting, failure to thrive, dyspnea, diaphoresis, arm and abdominal pain, fainting, recurrent fever, bronchitis, pneumonia, autoimmune chronic thyroiditis, Gilbert syndrome, and left corneal transplantation for corneal ectasia.	c.1334delG/p.(Gly445GlufsTer40)	Frameshift	HM	P	Cotti Piccinelli et al. 2021 [12]
P15	M/6 years	Clinical cerebrovascular event NR. Moderate generalized hypotonia and signs of denervation on electromyography/electroneurography.	Tortuosity and elongation of the aortic arch, aorta, supra-aortic trunks, brachiocephalic vessels, celiac trunk, superior mesenteric, renal, vertebral, subclavian, axillary, and brachial arteries. Compression of the inferior vena cava was reported.	MRA showed cerebral vascular loops and multiple elongations/loops in the Willis polygon, without arteriovascular malformations or aneurysms.	Hyperlaxity, elongated face, high-arched palate, beaked nose, micro-retrognathia, elongated philtrum, hyperextensible skin, mild scoliosis, patellar hyperlaxity, and genu recurvatum.	c.1334delG/p.(Gly445GlufsTer40)	Frameshift	HM	P	Palanca Arias et al. 2022 [13] (Patient 1/Family A)
P16	F/17 months	Mild motor developmental delay and mild axial hypotonia. Clinical stroke or seizure NR. EEG at 19 months showed symmetric bihemispheric moderate slowing without epileptiform discharges.	Pulmonary artery stenosis and diminutive descending aorta. Marked arterial tortuosity throughout the neck and head. No aortic tortuosity was reported.	MRI/MRA showed marked tortuosity in all cerebrovascular distributions, including anterior, middle, and posterior cerebral arteries, as well as basilar and vertebral arteries with a corkscrew appearance. No stroke, aneurysm, or stenosis was detected.	Term birth, elongated and narrow facies, drooping cheeks, blepharophimosis, down-slanting palpebral fissures, mild dolichocephaly, micro-retrognathia, large ears, arachnodactyly, and keratoconus. High-arched palate, beaked nose, and pectus deformity were not observed.	c.739C > A/p.(Gln247Lys), c.1162C > T/p.(Arg388Trp)	Missense + missense	HM; both variants reported homozygous	VUS, Conflicting interpretations of pathogenicity	Tempchin et al. 2022 [14]
P17	M/7 years, brain MRI at 1 year	Clinical cerebrovascular event NR. Microcephaly and mild white matter loss on brain MRI were reported, with subsequent normal neurodevelopment.	Mild aortic root dilation. Tortuosity of the aortic root and aortic arch, mainly ascending aorta. Tortuosity of carotid and vertebral branches, subclavian, humeral, arm and forearm vessels, and pulmonary arteries. Arterial stenosis, cardiomyopathy, and pulmonary arterial hypertension were absent.	Brain MRI revealed vascular tortuosity of the vertebrobasilar circulation and circle of Willis, tortuous and dysplastic internal carotid arteries, and leptomeningeal collaterals.	Term birth, complex uropathy with severe bilateral pyelectasis, non-obstructive non-refluxing megaureter, cryptorchidism, skin hyperextensibility, characteristic facial features, inguinal and diaphragmatic hernia, mild asymptomatic esophageal dilatation, severe myopia, and intermittent angle exotropia.	c.510G > A/p.(Trp170Ter)	Nonsense	HM	P	Esmel-Vilomara et al. 2023 [15] (Patient 1/Family 1)
P18	F/6 years	Clinical cerebrovascular event NR. Mild hypoplasia of the corpus callosum was reported in neonatal age.	Tortuosity predominantly involved the vertebral arteries, transverse aortic arch, descending aorta, common-external iliac arteries, and posterior tibial arteries. Arterial stenosis, cardiomyopathy, and pulmonary arterial hypertension were absent.	MRI angiography showed cerebrovascular arterial tortuosity involving arteries of the carotid and vertebrobasilar circulation.	Low-set ears, hypertelorism, down-slanting palpebral fissures, high narrow palate, prominent forehead, sagging cheeks, and joint hypermobility.	c.510G > A/p.(Trp170Ter)	Nonsense	HM	P	Esmel-Vilomara et al. 2023 [15] (Patient 2/Family 1)
P19	F/14 years, diagnosis at 11 years	Clinical cerebrovascular event NR.	Predominant supra-aortic arterial tortuosity involving vertebral, carotid, and subclavian arteries. Arterial stenosis, cardiomyopathy, and pulmonary arterial hypertension were absent.	MRI angiography showed cerebrovascular arterial tortuosity especially involving the carotid circulation with loops, middle and anterior cerebral arteries, and vertebrobasilar circulation.	Low-set ears, hypertelorism, down-slanting palpebral fissures, high narrow palate, hypoplasia of facial musculature, high anterior hairline, joint hypermobility, pleural effusion, and pulmonary hypoplasia that resolved spontaneously.	c.510G > A/p.(Trp170Ter)	Nonsense	HM	P	Esmel-Vilomara et al. 2023 [15] (Patient 3/Family 1)
P20	M/6 years, diagnosis at 2 years	Clinical cerebrovascular event NR. Normal neurological development, except dyslalia improved with speech therapy.	Very elongated and tortuous aortic root and arch with bending causing mild stenosis. Tortuosity of supra-aortic trunks, pulmonary arteries, upper extremity, iliac, and femoral arteries. Focal stenosis at the origin of the left renal artery. Right pulmonary artery kinked at the origin. Cardiomyopathy and pulmonary arterial hypertension were absent.	MRI angiography showed loops in both carotid and circle of Willis arteries, prominent posterior communicating arteries, right vertebral dominance, and left vertebral hypoplasia.	Diaphragmatic hernia, marked pectus excavatum, low-set ears, hypertelorism, down-slanting palpebral fissures, high narrow palate, joint hypermobility, severe myopia, and myopic astigmatism.	c.417T > A/p.(Tyr139Ter), c.899T > G/p.(Leu300Trp)	Nonsense + missense	CH	P/LP, P/LP	Esmel-Vilomara et al. 2023 [15] (Patient 4/Family 2)
P21	F/12 months, prenatally detected at 21 gestational weeks	Clinical cerebrovascular event NR. At 12 months, the infant was asymptomatic, alive, growing, and developing well.	Prenatally detected diffuse arterial tortuosity at 21 weeks. Fetal echocardiography showed severe tortuosity and elongation of the aortic arch, ductal arch, and proximal branch pulmonary arteries. Postnatal echocardiography confirmed severe aortic arch tortuosity without coarctation. Mild branch pulmonary artery stenosis was reported. Small aortopulmonary collateral vessels from the head and neck vessels and proximal descending aorta were present. Whole-body CT angiography showed profound tortuosity of the aorta, pulmonary arteries, iliac vessels, and common femoral arteries.	Whole-body CT angiography showed middle cerebral artery tortuosity. Vertebral tortuosity index was 81.	Delivered at 35 6/7 weeks by cesarean section due to severe preeclampsia. Brief continuous positive airway pressure support was required for transient tachypnea of the newborn. No hypertension, no medications, and multidisciplinary follow-up were reported at 12 months.	c.173C > T/p.(Ala58Val)	Missense	HM	VUS	Tunks et al. 2025 [16]
P22	F/12 months	Postoperative neurological deterioration after hiatal hernia repair, with loss of previously acquired developmental skills. Delayed neuromotor development, limited eye tracking, loss of expressive smile, inability to sit independently, and absence of clinically meaningful words at last follow-up.	Tortuous aortic arch. Ventricular septal defect, peripheral pulmonary stenosis, patent foramen ovale, and persistent left superior vena cava. CT angiography showed severe tortuosity of the aortic arch involving all major branches and the descending aorta.	Diffusion MRI showed diffusion restriction across nearly all cortical structures, compatible with hypoxic–ischemic encephalopathy. Brain MR angiography showed cerebral vessel tortuosity with distal branch narrowing in the MCA, ACA, and PCA territories.	Term birth to first-degree cousin parents. Familial history of congenital heart disease, including a sibling who died of tetralogy of Fallot. Long face, long philtrum, micrognathia, high-arched palate, increased skin elasticity, joint laxity, type III hiatal hernia, and bilateral inguinal hernias containing ovarian tissue.	c.485G > A/p.(Trp162Ter)	Nonsense	HM	P(PVS1,PM2,PP5)	Our case

PN: patient number; M: male; F: female; ATS: arterial tortuosity syndrome; MRA: magnetic resonance angiography; MRI: magnetic resonance imaging; CT: computed tomography; CS: clinical significance; EEG: electroencephalography; MCA: middle cerebral artery; ACA: anterior cerebral artery; PCA: posterior cerebral artery; HM: homozygous; CH: compound heterozygous; P: pathogenic; LP: likely pathogenic; P/LP: pathogenic/likely pathogenic; VUS: variant of uncertain significance; NR: not reported/not available.

**Table 2:** Docking and MM/GBSA scores of nine reported GLUT10 ligands for wild and mutant types.

	Docking Score (kcal/mol)		MM/GBSA Score (kcal/mol)	
Pubchem ID	Wild Type	Mutant Type	Wild Type	Mutant Type
5984	−6.458	−4.013	−36.56	−19.66
892	−7.469	−3.654	−34.46	−14.7
439213	−7.394	−3.552	−24.37	−14.52
18950	−7.305	−3.962	−28.28	−13.05
5793	−7.134	−3.947	−28.26	−17.16
16219248	−6.891	−3.747	−28.06	−14.69
6036	−6.702	−3.492	−24.42	−16.78
9815229	−6.516	−2.983	−22.98	−12.89
440667	−5.772	−3.323	−18.84	−18.7

## Data Availability

The original contributions presented in this study are included in the article material. Further inquiries can be directed to the corresponding author.

## Abbreviations

The following abbreviations are used in this manuscript:

ACA	Anterior Cerebral Artery
ATS	Arterial Tortuosity Syndrome
cDNA	Complementary DNA
CH	Compound Heterozygous
CT	Computed Tomography
EEG	Electroencephalography
F	Female
HM	Homozygous
LP	Likely Pathogenic
M	Male
MCA	Middle Cerebral Artery
MRA	Magnetic Resonance Angiography
MRI	Magnetic Resonance Imaging
NMD	Nonsense-Mediated mRNA Decay
NR	Not Reported/Not Available
P	Pathogenic
P/LP	Pathogenic/Likely Pathogenic
PCA	Posterior Cerebral Artery
PN	Patient Number
TMHMM	Transmembrane Helices in Proteins Analysis
VUS	Variant of Uncertain Significance
WES	Whole Exome Sequencing
